# Pregnancy rate and time to pregnancy after recurrent implantation failure (RIF)—a prospective cohort follow-up study

**DOI:** 10.1007/s10815-024-03257-9

**Published:** 2024-09-30

**Authors:** Linda B. P. M. Stevens Brentjens, Relinde J. E. Roumen, Luc Smits, Josien Derhaag, Andrea Romano, Ron J. T. van Golde, Janneke E. den Hartog

**Affiliations:** 1 Department of Obstetrics and Gynaecology, Maastricht University Medical Center+, P. Debyelaan 25, 6229HX Maastricht, The Netherlands; 2 GROW Research Institute for Oncology and Reproduction, Maastricht University, Maastricht, The Netherlands; 3 Department of Epidemiology, CAPHRI Care and Public Health Research Institute, Maastricht University, P. Debyelaan 25, 6229HX Maastricht, The Netherlands

**Keywords:** IVF, ICSI, Pregnancy rate, Recurrent implantation failure

## Abstract

**Purpose:**

The goal of this study was to determine ongoing pregnancy rate, time to pregnancy and embryo transfers to pregnancy within a cohort of patients with recurrent implantation failure (RIF).

**Methods:**

IVF patients with RIF were included after referral to the RIF outpatient clinic. They received a questionnaire 1 year after inclusion. If data was missing, medical files were examined to determine pregnancy outcomes and conception methods. The ability of the RIF outpatient clinic to improve pregnancy chance or increase the number of patients who elected to continue treatment was beyond the scope of this study.

**Results:**

The cumulative incidence of ongoing pregnancy in IVF patients with RIF (*n* = 79) after 1 year of follow-up was 40.5% (95% confidence interval = 30.4–51.5%). Median time to pregnancy was 4 months. Pregnancy incidence increased gradually up to 5 embryo transfers (mostly single embryo transfers). The average embryo transfers to pregnancy were 7.3 transfers.

**Conclusion:**

In IVF patients with RIF, up until the 5th embryo transfer, each transfer represents a good opportunity for ongoing pregnancy. This data can be used to counsel patients that regular treatment continuation seems to be well justified even when IVF patients fulfil the RIF criteria.

**Trial registration:**

CCMO: NL66835.068.18. METC 18–040. OMON: NL-OMON24778

**Supplementary Information:**

The online version contains supplementary material available at 10.1007/s10815-024-03257-9.

## Introduction

Infertility currently affects approximately 48 million couples worldwide [1]. Medical assisted reproduction (MAR) such as in vitro fertilization (IVF) and intracytoplasmic sperm injection (ICSI) have advanced in the last decades, but despite wide-ranging developments in ART many couples still fail to conceive. Implantation is considered one of the bottlenecks of treatment success. During implantation, a viable blastocyst has to appose, adhere and invade into the receptive endometrium of the uterus. This interaction can only occur after both blastocyst and endometrium have synchronically undergone structural and functional changes during the window of implantation, day 19 to 21 of a regular menstrual cycle [2, 3].

In 2021, the implantation rates in the Netherlands per embryo transfer were 27.6% for IVF, 33.6% for ICSI and 28.2% for frozen embryos (either IVF or ICSI) [4]. An estimated portion of 10–15% of the IVF patients experience recurrent implantation failure (RIF), although this percentage varies as no consensus concerning the definition has yet been reached [5, 6]. Up until recently, RIF was mostly defined as having at least 3 failed embryo transfers, with “failed transfers” being defined as transfers not leading to implantation nor a positive pregnancy test, with high-quality embryos, or the failed transfer of 10 or more embryos in multiple transfers [7]. However, multiple elements of the RIF definition are subject of discussion, such as the number of transferred embryos, embryo quality, number of IVF cycles, method of defining pregnancy and maternal age [8]. Due to the lack of consensus regarding the definition of RIF, it is difficult to compare data and determine an accurate reproductive prognosis of RIF patients. The European Society of Human Reproduction and Embryology (ESHRE) good practice recommendation on RIF has now identified RIF based on the chance of successful implantation for the individual patient and stated that further investigations and/or inventions are warranted if viable embryos failed to implant sufficiently often.

The aetiology of implantation failure is complex and multifactorial. Parental factors such as age, high BMI, tobacco or alcohol intake, stress, immunological factors, sperm quality and uterine factors have been described [5, 9–11]. However, with the knowledge gained by relatively novel technologies such as Pre-implantation Genetic Testing for aneuploidy (PGT-A), the reason for implantation failure is more often pointing towards the embryo [12, 13].

RIF causes anxiety, elevated stress levels and lowered quality of life in IVF patients [14–16]. Affected couples are often desperate and demand further diagnostic evaluations or endometrial testing to investigate and influence RIF. This is a challenge for clinicians, as a “keep calm and carry on” policy might be the most defensible approach currently available, but on the other hand, they can well understand the urge that is felt by the patient [17]. Being able to give patients insight in the chances of achieving pregnancy after the phenomenon of RIF would be helpful to mitigate the experienced stress. A retrospective follow-up study described a cumulative incidence of live birth in 118 RIF patients of 49% after a maximum follow-up period of 5.5 years and 25% after 1-year follow-up [18]. The present study explored pregnancy rate and time to pregnancy in IVF patients with RIF who were referred to a specialized RIF outpatient clinic as part of a prospective follow-up study [19], to be able to improve counselling strategies for RIF patients and to offer insight in corresponding prognosis.

## Materials and methods

### Ethical approval

The study was ethically approved by the Medical Research Involving Human Subjects Act of the Maastricht University Medical Center + (MUMC +) and Maastricht University (UM) (NL66835.068.18/METC 18–040) and registered prospectively in the Dutch Trial Register (CCMO Onderzoek met mensen, https://onderzoekmetmensen.nl/nl/trial/56176).

### Study design

This prospective cohort follow-up study was part of the Multidisciplinary Research on Repeated Implantation Failure and Recurrent Miscarriages (MURIM) study. The study protocol has been published before [19]. Briefly, the MURIM study analyses the steroid profile, microbiome and immunology status in patients with RIF, recurrent miscarriages and fertile controls. Female patients visited the RIF outpatient clinic during the window of implantation, 5 to 8 days after a positive ovulation test. The visit included a questionnaire, BMI and blood pressure measurement, an endometrial biopsy (“scratch”), a vaginal ultrasound, the collection of vaginal mucus and peripheral blood. Primary infertility was defined as never having conceived before. Systolic blood pressure > 140 mmHg and/or diastolic blood pressure > 90 mmHg was considered as hypertension. The vaginal mucus was used to determine the vaginal microbiome via the ReceptIVFity test. The ReceptIVFity test is a predictive test to assess an individual’s suitability for embryo implantation based on the microbiome resulting in a low, medium or high profile in the upcoming two months [20]. The blood sample was used to determine hormone (oestradiol, progesterone, free thyroxine (fT4) and thyroid stimulating hormone (TSH)) and vitamin D levels. After visiting the RIF outpatient clinic, patients were followed up for 1 year to monitor subsequent fertility treatments as well as occurrence and outcome of pregnancy. Although treatment advices were given, the ability of the RIF outpatient clinic to improve pregnancy chance and increase the number of patients who elected to continue with treatment was beyond the scope of this study.

### Study participants

All IVF patients who were referred to the RIF outpatient clinic and were included in the MURIM study during the time period of April 2019 until September 2023 were selected as part of this follow-up study. The RIF group consisted of patients aged between 18 and 38 years old who were treated at the MUMC + or elsewhere in the Netherlands and were referred to the MUMC + for RIF. RIF was defined as implantation failure after three transfers of high-quality embryos or after transfer of ten or more embryos in multiple transfers [7]. High-quality embryos were defined using Gardner criteria. High quality was defined as (i) cleavage-stage embryos consisting of 4-cells (2 days following fertilization) or ≥ 7 cells (3 days following fertilization) both with less than 20% fragmentation and the absence of multinucleation, (ii) 4 days following fertilization: morula grade A or blastocyst grade B1 or B2, (iii) 5 or 6 days following fertilization: blastocyst grade B3 or more combined with inner cell mass quality A or B and trophectoderm epithelium quality A or B. Exclusion criteria for RIF were BMI > 35 kg/m^2^, clinically relevant intrauterine pathology, untreated endocrine abnormalities or pre-implantation genetic testing (PGT) treatment for monogenetic disorders or structural rearrangements (PGT-M or PGT-SR). PGT for aneuploidy (PGT-A) is not performed in the Netherlands because of the current legislation, so aneuploidy data of the embryos of the included patients were unknown.

Informed consent regarding a 1-year follow-up was obtained at the start of the MURIM study. Participants consented to receive a questionnaire and/or to have the medical file examined.

### RIF diagnosis and treatment

After visiting the RIF outpatient clinic, treatment advices were given based upon the clinical outcomes. Lifestyle advices were given in case of smoking, alcohol abuses or high caffeine intake (> 200 mg per day). Weight loss and physical activity were advised in case of a high BMI (> 25 kg/m^2^). If the endometrium was thin (< 7 mm) during the WOI, it was advised to monitor endometrial thickness with an ultrasound before the next embryo transfer. If the endometrium remained thin, it was advised to change treatment protocol (e.g. artificial frozen cycle with oestradiol to natural cycle or natural cycle to artificial frozen cycle with follicle stimulating hormone) to potentially increase endometrial thickness. If vitamin D was low (< 50 nmol/L), supplements (10 mcg vitamin D/day) were advised. In case of low progesterone (< 20 nmol/L), progesterone supplements were advised when cryopreserved embryos were transferred in a natural cycle. In case of thyroid dysfunction, patients were referred to their general practitioner for treatment. When the ReceptIVFity outcome was low, treatment was delayed with three months. The ReceptIVFity test was repeated after 3 months but even in case of another low profile the IVF attempt was resumed, as no treatment was available yet.

### Instruments and data collection

A questionnaire was used to obtain relevant information from the study participants. The questionnaire elicited information regarding (dis)continuation of fertility treatment after participation in the MURIM study as well as occurrence of ongoing pregnancy during the 1-year follow-up period. The eligible patients were invited to participate 1 year after their visit to the outpatient clinic to participate in the study and were sent a short questionnaire on paper or by email. Missing information was collected via medical files and patients were called if there was no reply by post or email. Clinical factors obtained at intake at the RIF outpatient clinic were used as baseline characteristics. Missing data is reported and not imputed.

### Control group and ESHRE working group definition

To compare cumulative ongoing pregnancy rate in IVF women with RIF with non-RIF patients, the centre-specific treatment-process planning tool was used to determine all eligible IVF patients that could be included in the control group. All patients who started their first IVF or ICSI treatment at the MUMC + between January 2022 and April 2023 were analysed until data of 100 patients was gathered. Patients were included in the control group if they were between 18 and 38 years old and had a BMI < 35 kg/m^2^. Then, 1:1 matched controls were selected, matched for age, primary vs. secondary infertility and BMI. At last, indication for the IVF/ICSI treatment, duration of infertility, amount of ETs in the follow-up period and ongoing pregnancy rate was determined in the first year after the start of their first attempt via their electronic patient file.

To compare how many of the included RIF patients complied with the recommendations on RIF that were proposed by the ESHRE Working Group in 2023 [21], we used the recommended threshold of 60% predicted cumulative chance of implantation to identify RIF as defined by the working group based on the European IVF Monitoring Programme data [22].

### Statistical analysis

The primary outcome measures were incidence of ongoing pregnancy and time to pregnancy within 1 year after participation in the MURIM study. Time to pregnancy was calculated as time in months between visiting the RIF outpatient clinic and the date of the first positive pregnancy test which eventually led to a pregnancy lasting at least 12 weeks. Secondary outcome measures were (i) amount of embryo transfers to achieve ongoing pregnancy within one year after visiting the RIF outpatient clinic (“transfers to pregnancy”), (ii) method of conception (fresh or frozen embryo transfer), (iii) pregnancy outcomes and (iv) influence of baseline determinants on pregnancy chance. Normal distribution of baseline characteristics was assumed based on the amount of participants [23]. Baseline characteristics were compared between patients who did and did not achieve ongoing pregnancy within the 1-year follow-up period using univariable analysis. Continuous variables were compared using an independent Student’s *T*-test. For categorical variables, risk ratios (RR) were calculated to compare exposure in non-pregnant vs. pregnant patients using OpenEpi version 3.01 (www.OpenEpi.com). The Statistical Package for the Social Sciences (SPSS v 22 for Windows, Chicago, IL, USA) was also used for data analysis. A *p*-value below 0.05 was considered statistically significant. Data are presented as mean (± SD) or number (%). Time to pregnancy is shown as median values with interquartile range (IQR). Transfers to pregnancy are presented as mean with 95% confidence intervals (CI).

Time to pregnancy and number of transfers to pregnancy were estimated by one minus Kaplan–Meier (KM) survival curves. Time to pregnancy does not take into account that couples might choose for a break in their treatment and have very little chances during that month (as spontaneous pregnancy chances in RIF patients are assumed to be low). Therefore, embryo transfers to pregnancy are calculated as they are more informative in a clinical setting. Censoring of data was applied in two different ways to determine transfer to pregnancy survival curves. Typically, in KM curves, patients are censored if they stop further treatment during the follow-up period (“optimistic curve”). In the optimistic scenario, it is assumed that patients who stopped treatment had the same probability of achieving an ongoing pregnancy after IVF/ICSI as those who continued. However, to avoid overestimation of pregnancy chances, a second curve was calculated (“pessimistic curve”) which accounts for the scenario that the participant who stopped treatment was advised to do so because of a poor prognosis. The pessimistic curve assumes that patients who stopped treatment without achieving pregnancy had no more chance of achieving pregnancy [24]. In this scenario, the amount of patients at risk during the embryo transfer was used to calculate the cumulative incidence of ongoing pregnancy for all subsequent embryo transfers, regardless of the reason why they stopped further treatment. The transfer could have either implied a transfer of one or two embryos.

## Results

### Pregnancy rate and clinical characteristics

**Fig. 1:**
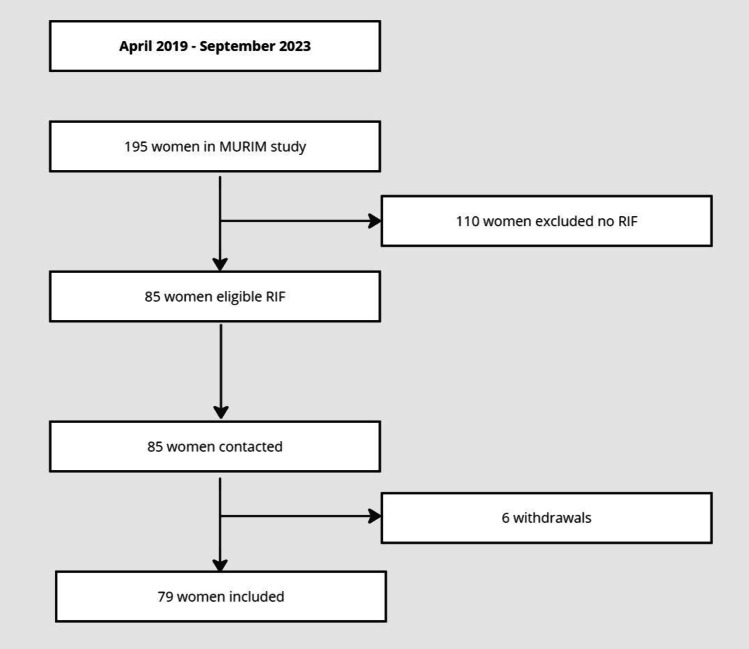
Flowchart. Flowchart showing inclusion of RIF patients from recruitment in MURIM study towards selection for this study

A total of 195 patients participated in the MURIM study during the time period of April 2019 until September 2023. Of these, 85 participants were included in the follow-up study after visiting the RIF outpatient clinic. The remaining patients of the MURIM study had either recurrent miscarriages or were fertile controls. Six participants were excluded from the follow-up study as they chose to withdraw. Data was collected from 79 patients (response rate = 92.9%) (Fig. 1). Patients had a mean age of 33.8 ± 3.1 years and infertility duration of 40.4 ± 25.9 months upon inclusion. The average amount of previous ovum pick ups was 2.2 ± 0.8 with an average of 7.1 ± 2.7 embryo transfers (range 3–14). Main causes of infertility were male factor (46.8%), idiopathic (29.1%) and tubal pathology (17.7%). Other causes were endometriosis (2.5%), male factor combined with tubal pathology (2.5%) and tubal pathology combined with endometriosis (1.3%). A total of 58.2% patients had primary infertility. Mean BMI was 25.7 ± 4.1 kg/m^2^, 93.6% of patients were non-smokers and ethnicity was Caucasian (87.3%), Middle-East/North-African (10.1%) and Asian (2.5%). When comparing the criteria for RIF from this study with the ESHRE working group on RIF recommendations, 97.5% (*n* = 77) of our patients also comply with the RIF criteria according to the working group.

At the end of the follow-up period, 32 out of 79 IVF patients with RIF achieved an ongoing pregnancy (40.5%, 95% confidence interval [CI] = 30.4–51.5%). Baseline characteristics between the IVF patients with RIF who achieved an ongoing pregnancy during the follow-up vs. IVF patients with RIF with no pregnancy during the follow-up period were similar (Table 1). To interpret the RIF data with non-RIF patients undergoing similar treatment, a matched control group undergoing their first IVF or ICSI treatment was included (see Methods). A total of 79 women were included with an average age of 33.3 ± 3.1 years, primary infertility rate of 64.6% and BMI of 25.3 ± 4.2 kg/m^2^. Duration of infertility was 35.7 months, comparable to the RIF group and the main difference was the presence of PCOS/ovulatory dysfunction as an indication for infertility. AMH levels were similar between the two groups. The control group had an average of 2.2 ± 1.6 embryo transfers in the year after the start of their first IVF or ICSI treatment. In comparison with this non-RIF control group, ongoing pregnancy rate was significantly lower in the RIF population (70.9% vs. 40.5% respectively, *p* < 0.01, by the chi-square test). A comprehensive overview of the baseline characteristics and treatment outcome of the non-RIF control group are given in Supplementary Table 1.

**Table 1:** Baseline characteristics of RIF patients who did and did not achieve ongoing pregnancy within the 12-month follow-up period

	Ongoing pregnancy (*n* = 32)	No pregnancy (*n* = 47)	*P*-value
Age at inclusion (years)	33.7 ± 3.7	33.9 ± 2.7	0.77
Obesity (BMI > 30 kg/m^2^)	4 (12.5)	13 (27.7)	NS
Hypertension^a^	7 (23.3)	4 (8.7)	NS
Primary infertility	14 (43.8)	19 (40.4)	NS
Duration of infertility (months)	40.6 ± 28.8	40.2 ± 24.1	0.96
Indication for IVF/ICSI			
Male	16 (50)	21 (44.7)	NS
Idiopathic	10 (31.3)	13 (27.7)	NS
Tubal pathology	5 (15.6)	9 (19.1)	NS
Endometriosis	0 (0)	2 (4.3)	NS
Tubal factor and endometriosis	0 (0)	1 (2.1)	NS
Male factor and tubal factor	1 (3.1)	1 (2.1)	NS
No. of embryos transferred before inclusion^b^	7.3 ± 2.5	7.0 ± 2.8	0.57
Length of luteal phase (days)^c^	11.6 ± 1.9	11.9 ± 2.8	0.62
Endometrial thickness (mm)^d^	8.6 ± 3.2	8.8 ± 2.6	0.79
Progesterone (nmol/L)^e^	24.3 ± 9.4	26.9 ± 7.8	0.18
Oestradiol (nmol/L)^f^	0.52 ± 0.24	0.47 ± 0.17	0.29
TSH (mU/L)	2.1 ± 1.5	1.9 ± 0.8	0.47
AMH^g^	3.28 ± 1.88	3.08 ± 2.37	0.69
Vitamin D^h^	64.7 ± 20.5	66.5 ± 21.4	0.71
Low ReceptIVFity test	7 (21.9)	15 (31.9)	NS

Data are presented as mean (± SD) or number (%). Associations are calculated univariable because of low event number. *NS*, non-significant risk ratio. *BMI*, body mass index, hypertension: systolic blood pressure > 140 mmHg and/or diastolic blood pressure > 90 mmHg, ReceptIVFity: predictive test to assess an individual’s suitability for embryo implantation based on the microbiome in the upcoming two months (20). ^a^Blood pressure data was missing for 2 pregnant patients. ^b^The number of embryo transfers in the current treatment (previous transfers which led to an ongoing pregnancy were excluded). ^c^Length of the luteal phase was missing for 16 non-pregnant and 5 pregnant patients. ^d^Endometrial thickness was missing for 1 non-pregnant and 1 pregnant patient. ^e^Progesterone was missing for 1 non-pregnant patient. ^f^Oestradiol was missing for 1 pregnant and 1 non-pregnant patient. ^g^AMH was determined upon inclusion or from medical files. AMH was missing for 1 non-pregnant patient. ^h^Vitamin D was missing for 2 non-pregnant patients

### Time and transfers to pregnancy

**Fig. 2:**
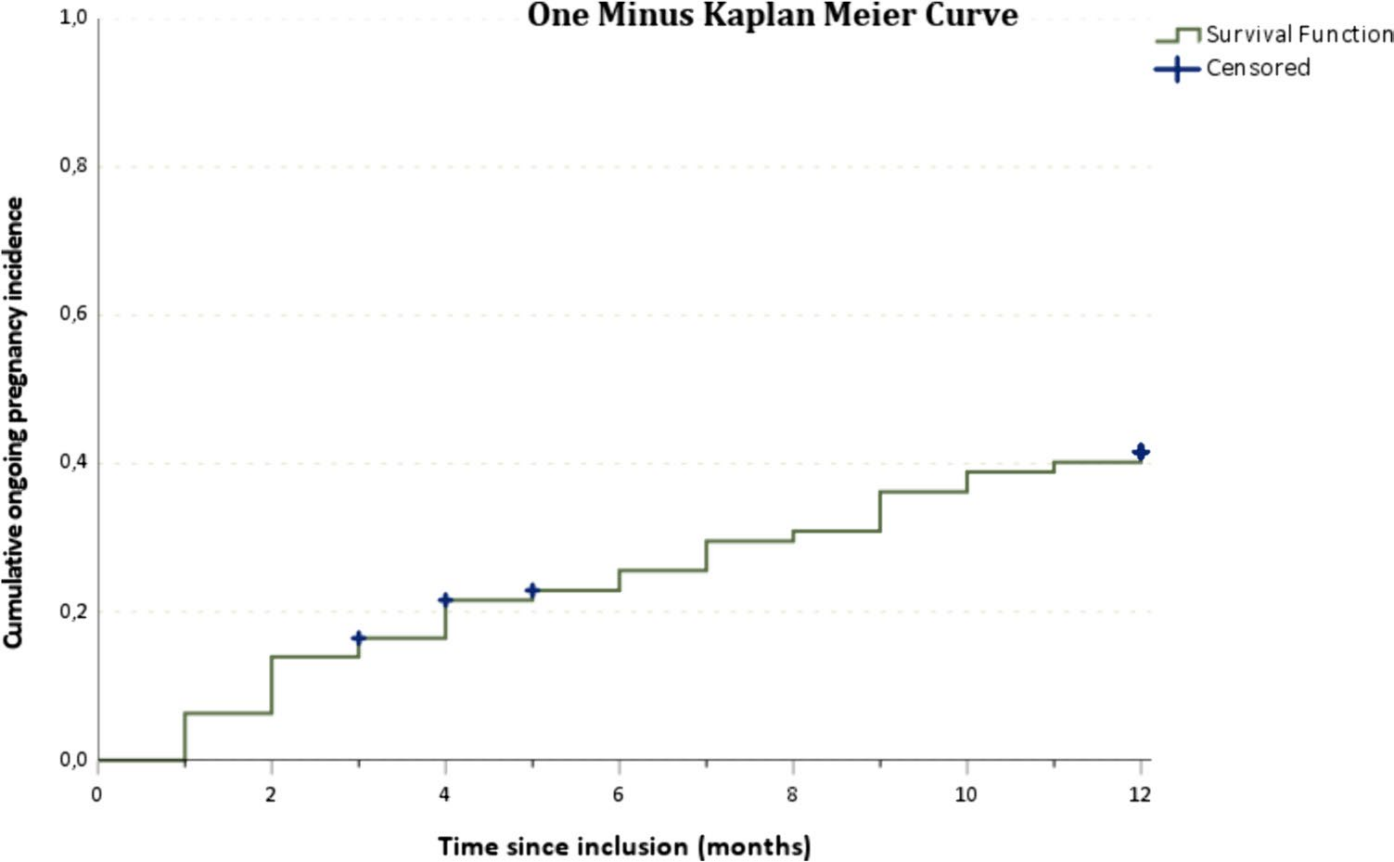
Kaplan–Meier curve time to pregnancy. One minus Kaplan–Meier curve showing the amount of RIF patients with an ongoing pregnancy during the follow-up period of 12 months

**Fig. 3:**
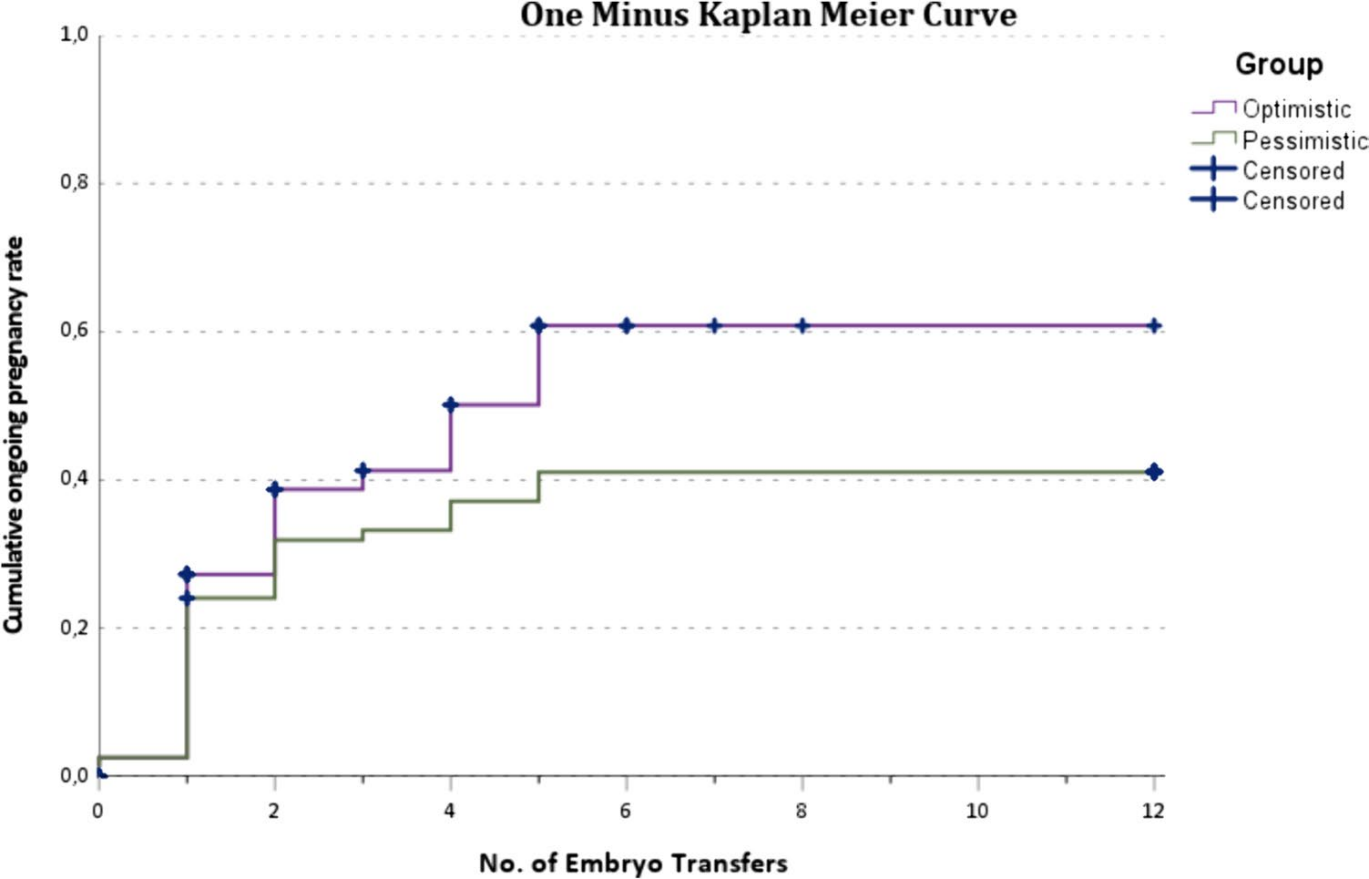
Kaplan–Meier curve transfers to pregnancy. One minus Kaplan–Meier curve showing (i) the optimistic scenario in which a patient was censored when she had no more embryo transferred and (ii) the pessimistic scenario in which a patient remained at risk

IVF patients with RIF with an ongoing pregnancy in the follow-up period (*n* = 32) had a median time to pregnancy of 4 months (IQR = 2–8.75). Pregnancy incidence increased gradually during the follow-up period without ceiling effect as is visualised in the Kaplan–Meier curve (Fig. 2). Within 1 year after visiting the RIF outpatient clinic, between 0 and 12 embryo transfers were performed (mean = 2.3 ± 2.2). Pregnancy incidence based on the amount of embryo transfers is shown in a Kaplan–Meier curve (Fig. 3). In the optimistic curve, patients are censored if there were no more transfers. In the pessimistic curve, patients remained at risk when there were no more transfers, i.e. the chance of pregnancy is assumed zero if there would have been more transfers (see Methods). Both scenarios show a gradual increase in pregnancy incidence up until the fifth embryo transfer, after which a plateau phase is reached. The overall estimate number of embryo transfers to reach ongoing pregnancy was 7.3 transfers (95% CI 6.4–8.1). In the optimistic scenario, mean transfer to pregnancy was 6.1 transfers (95% CI 4.7–7.5). In the pessimistic scenario, mean transfer to pregnancy was 7.8 transfers (95% CI 6.7–9.0).

### Method of conception and pregnancy outcomes

Most pregnancies arose from a transfer of a single cryopreserved embryo (*n* = 17, 53.1%). One patient gave birth to dizygotic twins which arose from a cryopreserved single embryo transfer and a spontaneous pregnancy simultaneously. Despite RIF, two pregnancies occurred after a spontaneous conception (Supplementary Table 2). After 1 year of follow-up, the 32 ongoing pregnancies have led to 21 singletons (65.6%) and three twins (9.4%). Four pregnancies were still ongoing (12.5%), one led to a late miscarriage (gestational age 15 weeks, 3.1%) and two to a stillbirth (6.3%) (Supplementary Table 3).

### Diagnosis and treatment of RIF

After visiting the RIF clinic, clinical findings and treatment advices were given to the patients (see Methods). No implantation limiting factors were found in 19 patients (24.1%). In the remaining patients, one or more treatment advices were given. Weight loss and physical activity (because of a BMI > 25 kg/m^2^) was the advice that was given most often (31.6%). Next was delaying the next embryo transfer with 3 months (26.6%) based on a low ReceptIVFity test. Also, supplementing vitamin D was advised (19%) as well as progesterone supplementation (in frozen, natural cycles, 200 mg three times a day, 19%). Furthermore, evaluation of endometrial thickness was recommended (13.9%), smoking and alcohol advices (12.7%) as well as thyroid disease and treatment evaluation (6.3%) (see Table 2). Effectiveness of the clinic’s advices could not be determined as (i) there was no monitoring of treatment adherence and (ii) there was no control group available of RIF patients who did not visit the outpatient clinic. However, the risk of no pregnancy at the end of the follow-up treatment was not statistically significantly different in the non-treated RIF patients (*n* = 19), where no known implantation limiting factors were found, as compared to the RIF patients who received treatment because of implantation limiting factors (*n* = 60) (52.6% vs. 61.7% respectively, RR 0.91, 95% CI 0.70–1.19).

**Table 2:** Advices given based upon the findings of the RIF outpatient clinic

	Amount of patients (%)
Weight loss and physical activity	25 (31.6)
Delay treatment^a^	21 (26.6)
Vitamin D supplementation	15 (19)
Progesterone supplementation	15 (19)
Endometrial evaluation	11 (13.9)
Lifestyle advice	10 (12.7)
Thyroid function evaluation	5 (6.3)

^a^Treatment delay of three months was advised based on a low ReceptIVFity profile

## Discussion

This study determined the incidence of ongoing pregnancy within one year in 79 IVF patients who fulfilled the RIF criteria. A maximum of 12 embryo transfers were performed during the follow-up period with a high ongoing pregnancy rate of 40.5% (95% CI = 30.4–51.5). Most pregnancies were achieved by transferring a single cryopreserved embryo. The average transfers to pregnancy were 6.1–7.8 transfers (optimistic vs. pessimistic scenario). Pregnant patients had a median time to pregnancy of 4 months. Although the 1-year probability of pregnancy has been studied before in IVF or ICSI patients [25], less is known about the prognosis of patients with RIF. A complicating factor is that up until recently, no consensus regarding the definition of RIF was reached. One previous retrospective study by Koot et al. screened medical files to analyse pregnancy rates in IVF patients with RIF (defined as three sequential failed IVF or ICSI attempts with at least one good quality embryo transferred or with a total of 10 failed good quality transfers including frozen-thawed embryos). In this study, 49% of RIF patients had a live birth during the maximum of 5.5-year follow-up. After 1 year of follow-up, 25% of IVF patients with RIF had a live birth. Another study examined medical files respectively and found a live birth rate of 25.8% in the group of RIF patients with > 3 failed embryo implantations [26]. Both pregnancy rates were lower than the pregnancy rate found in our study. The difference in pregnancy rate could be explained by the difference in inclusion criteria and the outcome (ongoing pregnancy vs. live birth). Also, the described studies screened for RIF retrospectively by consulting medical files and no advices that could have led to increased pregnancy chances were given [18].

The results of the current study show that IVF patients with RIF have a lower 1-year pregnancy chance compared to the matched non-RIF control group consisting of women who started their IVF or ICSI treatment. Logically, IVF patients with RIF have a poorer prognosis compared to patients who start IVF/ICSI treatment as it is known that pregnancy chances per transfer decline when more embryos have been transferred in the past without success [26]. However, pregnancy chances in IVF patients with RIF still remained adequate and higher than expected during the first year after visiting the specialized outpatient clinic. Overall, the RIF cohort reflected the non-RIF control group, although some differences were found in the baseline criteria. Remarkably, duration of infertility upon inclusion was only 5 months longer in the RIF cohort compared to the control group. This was unexpected, as RIF patients had already 7 embryos transferred before inclusion, whereas, in the non-RIF group, no embryos were transferred so far. A tentative explanation is that couples who wait a long time before choosing to undergo an IVF/ICSI treatment may decide not to undergo further treatment and/or visit the specialized RIF clinic after treatment failure. In the non-RIF group, 17.7% of patients had a type of ovulatory dysfunction (PCOS and non-PCOS), whereas, in the RIF group, no ovulatory dysfunction was present. However, this cannot be related to the aetiology of RIF, as patients with ovulatory dysfunction were excluded in the MURIM study (as an endometrial biopsy had to be performed in the luteal phase of a natural cycle). We cannot rule out that exclusion of patients with ovulatory dysfunction impacts the 1-year ongoing pregnancy rate in IVF patients with RIF, although similar pregnancy rates were found between IVF women with and without PCOS [27].

It should be noted that overestimation of pregnancy chances cannot be fully excluded because (i) patients above 38 years old were excluded in this study, (ii) poor responders often do not reach the RIF criteria which can lead to exclusion of this group and (iii) only patients who were referred to the RIF clinic were included, which might cause an overestimation of pregnancy chances as patients with a poor prospective might not be referred. Nevertheless, the high pregnancy rate in RIF is reassuring. The high pregnancy rates in IVF patients with RIF raises the question whether the criteria that were used for RIF are accurate, as the high pregnancy rates also imply that at the time of RIF “diagnosis”, there was still a high chance of success. Possibly, a stricter definition of RIF could help in distinguishing patients who benefit from further investigations and interventions and patients who did not have enough embryo transfers yet to have an acceptable pregnancy chance. In the novel definition proposed by the ESHRE Working Group on RIF, ploidy status and maternal age are taken into account to determine the amount of unsuccessful previous embryo transfers [21]. When comparing the definition of RIF with embryos with unknown ploidy status with the definition used in this study, almost all patients (97.5%) complied. In our study, ploidy status of the embryo was unknown as PGT-A is currently forbidden in the Netherlands. It is described that when only euploid blastocyst are transferred, the cumulative live birth rate steadily increases to > 90% [13, 28]. Because of a possible improvement of the embryo factor after PGT-A after selecting chromosomally normal embryos for transfer, this could further impact pregnancy chances and time to pregnancy in IVF patients with RIF. Besides, improved opportunities to unravel the endometrial factor in RIF would arise if it would be possible to determine embryonic ploidy status in these patients.

To reach the high cumulative pregnancy incidence in this study, an average amount of seven embryo transfers was needed in patients with RIF during the 1-year follow-up period. Patients already had an average of two ovum pick ups upon inclusion, which implies that a third and possibly fourth IVF/ICSI attempt was performed in this cohort to have sufficient chances. In the Netherlands, a maximum of 3 IVF cycles are reimbursed. Thus, depending on each countries’ national health insurance policy, ovarian reserve of the patient and financial status of the couple, it might not always be possible to do so. Nevertheless, this study shows that especially up to the 5th transfer, each transfer represents a good opportunity for ongoing pregnancy. Therefore, patients can be counselled that, whenever the emotional and financial burden is bearable, additional attempts can be considered.

Strengths of this study are the prospective design and the high response rate. In this study, the novel definition “transfers to pregnancy” is introduced. As patients who have had multiple IVF/ICSI treatments may decide to take a treatment break for several months, time to pregnancy might be less informative. In this case, defining transfers to pregnancy is more relevant than time to pregnancy and we encourage experts in the field to include this term in their research outcomes. Because of the relatively small study size, multivariable analysis to determine underlying potential mechanisms of RIF was not possible. The main treatment advice that was given was related to a high BMI. Although it is well known that obesity is associated with infertility and implantation failure, the mechanism behind this is not fully understood [29]. Possibly, oocyte quality and follicular development might be affected in obesity [30]. Additionally, transcriptome analysis has revealed that pathways related to the immune response, inflammation and reactive oxygen species production were over-expressed in obese women, and that the window of implantation was displaced more often [31, 32].

Future multi-centre endeavours with prospective pregnancy data from a large sample of IVF patients with RIF are recommended to be able to perform multivariable analysis on aetiology. Additionally, further work is needed to determine the influence of treatment advices on pregnancy chance.

## Conclusion

In conclusion, this prospective follow-up study found a reassuringly high ongoing pregnancy rate of 40.5% during the 1-year follow-up in a sample of 79 IVF patients with RIF. The results of the current study can be used to counsel patients that treatment continuation seems to be well justified, even after a phenomenon of multiple failed transfers of viable embryos. However, in the decision-making process individual patient circumstances such as psychological and economic pressures should be considered, as well as the potential benefits and risks of possible interventions.

## Supplementary Information

Below is the link to the electronic supplementary material.

Supplementary file1 (DOCX 18.8 KB )

## Data Availability

Data is available upon request.
