# Supplementary material for: Pregnancy rate and time to pregnancy after recurrent implantation failure (RIF)—a prospective cohort follow-up study
Source: J Assist Reprod Genet. 2024 Sep 30;41(11):3061–70. doi: 10.1007/s10815-024-03257-9 (PMC11621263; doi:10.1007/s10815-024-03257-9)
Supplement: Supplementary file 1 — Supplementary file1 (DOCX 18.8 KB ) [file 10815_2024_3257_MOESM1_ESM.docx]

**Supplementary Table 1: Baseline characteristics and treatment outcome of the non-RIF control group**

|  | **Non-RIF control group (n=79)** |
| --- | --- |
| **Age at inclusion (years)** | 33.3 ± 3.1 |
| **Obesity (BMI > 30 kg/m^2^)** | 12 (15.2) |
| **Primary infertility** | 51 (64.6) |
| **Duration of infertility (months)** | 35.7 ± 21.5 |
| **Indication for IVF/ICSI** |  |
| **Male** | 26 (32.9) |
| **Idiopathic** | 18 (22.8) |
| **Tubal pathology** | 7 (8.9) |
| **Endometriosis** | 10 (12.7) |
| **Tubal factor and endometriosis** | 1 (1.3) |
| **Male factor and endometriosis** | 2 (1.3) |
| **Ovulatory dysfunction (non-PCOS)** | 3 (3.8) |
| **PCOS** | 5 (6.3) |
| **PCOS and endometriosis** | 1 (1.3) |
| **PCOS and male factor** | 3 (3.8) |
| **PCOS, endometriosis and male factor** | 2 (2.5) |
| **Uterine anomaly** | 1 (1.3) |
| **AMH^1^** | 3.4 ± 2.5 |
| **No. of embryos transferred during one-year follow up ^2^ (range)** | 2.2 ± 1.6 (0 – 6) |
| **Ongoing pregnancy during one-year follow up** | 56 (70.9) |

Data are presented as mean (±SD) or number (%). BMI: body mass index. ^1^AMH was determined before the start of the first IVF or ICSI treatment. ^2^ The number of embryo transfers in the year after the start of the first IVF or ICSI treatment (one-year follow up period).

**Supplementary Table 2: Method of conception for all ongoing pregnancies during the one-year follow-up period of the IVF patients with RIF**

|  | **Number of ongoing pregnancies (%)** |
| --- | --- |
| **Spontaneous** | 2 (6.3) |
| **Fresh SET** | 8 (25) |
| **Fresh DET** | 3 (9.4) |
| **Frozen SET** | 17 (53.1) |
| **Frozen DET** | 1 (3.1) |
| **Combined spontaneous + frozen SET** | 1 (3.1) |

SET: single embryo transfer, DET: double embryo transfer.

**Supplementary Table 3: Pregnancy outcome of all ongoing pregnancies after one-year follow-up**

|  | **Pregnancy outcome (%)^1^** |
| --- | --- |
| **Livebirth singleton** | 21 (65.6) |
| **Livebirth twins** | 3 (9.4) |
| **Ongoing pregnancy** | 4 (12.5) |
| **Miscarriage (late)^2^** | 1 (3.1) |
| **Stillbirth** | 2 (6.3) |

^1^Pregnancy outcome is missing from 1 patient with a spontaneous pregnancy. ^2^At a gestational age of 15 weeks
